# Fe(III) Ions-Assisted Aniline Polymerization Strategy to Nitrogen-Doped Carbon-Supported Bimetallic CoFeP Nanospheres as Efficient Bifunctional Electrocatalysts toward Overall Water Splitting

**DOI:** 10.3390/ma14061473

**Published:** 2021-03-17

**Authors:** Changhao Zhao, Fen Wei, Haolin Lv, Dengke Zhao, Nan Wang, Ligui Li, Nanwen Li, Xiufang Wang

**Affiliations:** 1 Guangdong Engineering & Technology Research Center of Topic Precise Drug Delivery System, School of Pharmacy, Guangdong Pharmaceutical University, 280 Waihuan Dong Road, University Town, Guangzhou 510006, China; tzzhaochanghao@163.com (C.Z.); wfweifen@163.com (F.W.); lyuhawlin@163.com (H.L.); 2 Guangzhou Key Laboratory for Surface Chemistry of Energy Materials, New Energy Research Institute, School of Environment and Energy, South China University of Technology, Guangzhou 510006, China; scutezhao@sina.com; 3 Guangdong Provincial Key Laboratory of Optical Fiber Sensing and Communications, Siyuan Laboratory, Guangzhou Key Laboratory of Vacuum Coating Technologies and New Energy Materials, Guangdong Provincial Engineering Technology Research Center of Vacuum Coating Technologies and New Energy Materials, Department of Physics, Jinan University, Guangzhou, Guangdong 510632, China; 4 Guangdong Provincial Key Laboratory of Advance Energy Storage Materials, South China University of Technology, Guangzhou 510640, China; 5 Key Laboratory of Coal Conversion, Institute of Coal Chemistry, Chinese Academy of Sciences, Taiyuan 030001, China; linanwen@sxicc.ac.cn

**Keywords:** overall water splitting, transition metal phosphide, nitrogen doped carbon, polyaniline, electrocatalyst

## Abstract

It remains an urgent demand and challenging task to design and fabricate efficient, stable, and inexpensive catalysts toward sustainable electrochemical water splitting for hydrogen production. Herein, we explored the use of Fe(III) ion-assisted aniline polymerization strategy to embed bimetallic CoFeP nanospheres into the nitrogen-doped porous carbon framework (referred CoFeP-NC). The as-prepared CoFeP-NC possesses excellent hydrogen evolution reaction (HER) performance with the small overpotential (η_10_) of 81 mV and 173 mV generated at a current density of 10 mA cm^−2^ in acidic and alkaline media, respectively. Additionally, it can also efficiently catalyze water oxidation (OER), which shows an ideal overpotential (η_10_) of 283 mV in alkaline electrolyte (pH = 14). The remarkable catalytic property of CoFeP-NC mainly stems from the strong synergetic effects of CoFeP nanospheres and carbon network. On the one hand, the interaction between the two can make better contact between the electrolyte and the catalyst, thereby providing a large number of available active sites. On the other hand, it can also form a network to offer better durability and electrical conductivity (8.64 × 10^−1^ S cm^−1^). This work demonstrates an efficient method to fabricate non-noble electrocatalyst towards overall water splitting, with great application prospect.

## 1. Introduction

With the increasing of the energy consumption and severe destruction of environment, green renewable energy is urgently needed to be developed and utilized [1,2] Hydrogen is confirmed to be the most reasonable and ideal energy carrier to replace fossil fuels [3,4,5]. Among many ways to produce hydrogen, electrochemical hydrolysis is considered to be the most desirable way and of great interest to researchers because of its high hydrogen purity, abundant source materials, and large-scale production [6,7]. Water splitting involves two important half reactions: Water oxidation (OER) and hydrogen evolution reaction (HER). Compared with HER, OER with a larger overpotential and sluggish kinetics will lead to a poor energy conversion efficiency, which is the reason why suitable catalysts are required [8,9,10]. So far, noble metal electrocatalysts based on Pt and Ru are still widely used as commercial electrocatalysts for HER and OER, respectively. Regrettably, big budgets and low reserves restrict their practical application [11,12,13]. Hence, developing an efficient, low-cost non-noble metal electrocatalyst has become an urgent need, and research interest in this field is growing [14,15]

Currently, researchers have made great progress in preparing non-noble metal electrocatalysts, among which transition metal oxides [16,17], hydroxides [18,19,20], selenides [21,22,23], phosphates [24], sulfides [25,26,27,28], and nitrides [29] have been widely explored. It is noteworthy that transition metal phosphide (TMPs) have attracted tremendous interest resulting from their high-activity and low-cost. Ma et al. reported a method to enhance HER by deposition of CoP nanoparticles on heteroatom-doped graphene, which exhibited good HER property in different types of electrolyte solutions [30]. Du and co-workers have synthesized bimetallic CoFeP hollow microspheres with good electrochemical performance, mainly due to the interaction of the CoP and FeP, which can adjust the electron density between those two metals, and thus greatly enhance the electrochemical activity [31]. However, TMPs has the disadvantage of inadequate electronic conductivity and accessible active sites [32,33]. Encouragingly, research shows that the introduction of carbon can effectively make up for the weakness [30,34]. Carbon can enhance electronic conductivity and increase the active area, while P can regulate the electronic structure and reduce the activation energy through electron interaction, whose combination can provide excellent electrocatalytic performance and stability [35,36,37]. Zhang’s group prepared core-shell structure of the hollow spheres which exhibited a low overpotential of 65 mV at η10 toward HER [38]. However, the following problem is that TMPs may agglomerate in carbon materials, which determines the active area and electrochemical properties. Meanwhile, the carbon coating thickness of TMPs material severely restrict electron transfer between TMPs and carbon as well as synergistic effect between them [34,39]. Based on hereinbefore problems, exploring state-of-art electrocatalysts is urgently needed.

Herein, we fabricated bimetallic CoFeP nanospheres which were embedded into nitrogen-doped porous carbon framework with the method of carbonation and phosphating treatment for CoFeO_x_-PANI precursor. PANI-derived carbon substrate can not only create network to offer high electronic conductive, increase active area between electrolyte and electrocatalysts, but also avoid the aggregation of CoFeP nanospheres aiming to greatly increasing utilization of active sites. Compared with the conductivity value of CoFeO_x_-PANI (2.15 × 10^−5^ S cm^−1^), CoFeP-NC has an excellent electrical conductivity of 8.64 × 10^−1^ S cm^−1^. In addition, the synergistic effect of CoFeP nanospheres and carbon network plays an active role in electrochemical performance. Notably, the as-prepared CoFeP-NC possessed overpotential of 81 and 173 mV for HER in acid and alkaline environment, respectively, and for OER, the value was 283 mV in 1 M KOH, which were comparable or even outstrips that of reported TMPs catalysts (Tables S1 and S2).

## 2. Experimental Section

### 2.1. Materials

Iron nitrate nonahydrate (Fe(NO_3_)_3_·9H_2_O), Cobalt nitrate hexahydrate (Co(NO_3_)_3_·6H_2_O), Sodium hypophosphite (NaH_2_PO_2_) and other chemicals are were all from Energy Chemicals of China (Guangzhou). Pt/C (20 wt%), RuO_2_ (99 wt%) and Nafion were purchased from Alfa Aesar (Shanghai, China). Aniline and ethanol were supplied by Aladdin Industrial Corporation (Shanghai, China). The resistivity of deionized water used in this experiment was 18.2 MΩ cm.

### 2.2. Synthesis of CoFeO_x_-PANI

CoFeO_x_-PANI was synthesized according to the method reported in the literature [40]. In brief, 1.212 g Fe(NO_3_)_3_·9H_2_O and 0.873 g Co(NO_3_)_3_·6H_2_O were first homogeneously mixed in the mixture of water and ethanol whose volume ratio was 1:1. Subsequently, the mixture was continuously stirred for 10 min to form a uniform solution. After 548 μL of aniline was added and vigorously stirred for 20 min, the solution was located at the sealed Teflon-lined autoclave, followed by heating to 200 °C for 720 min. After the obtained black precipitate was washed several times with deionized water and ethanol, the target product was collected by freeze drying.

### 2.3. Synthesis of CoFeP-NC

In a typical phosphating process, as-prepared CoFeO_x_-PANI precursor was placed in a porcelain boat, and then heat-treated under nitrogen protection at 900 °C for 120 min. The CoFe-NC intermediate was prepared through natural cooling to room temperature. Then, the obtained 30 mg CoFe-NC solid powder and 1 g sodium hypophosphite powder were placed in two separate porcelain boats, followed by heating to 400 °C for 180 min under N_2_ atmosphere. Subsequently, CoFeP-NC power was obtained. As a contrast, FeP-NC was obtained by the same procedure of CoFeP-NC, except for not adding Co(NO_3_)_3_·6H_2_O.

### 2.4. Characterization

The Powder X-ray diffraction (XRD) measurements were carried out on a Bruker D8 advance with a scanning rate of 2θ = 2° min^−1^ at 25 °C. X-ray photoelectron spectra (XPS) data were obtained on a Phi X-tool instrument with a monochromatic Al Kα source. The morphology and size of the materials were analyzed by transmission electron microscopy (TEM) and high-resolution TEM (HR-TEM) on a JEOL-2010 instrument (Tokyo, Japan). The ASAP 2010 instrument (ASAP 2010, Atlanta, GA, USA) were used to measure the specific surface area and corresponding pore size distribution of the products by nitrogen adsorption/desorption at 77 K.

### 2.5. Electrochemical Measurements

The electrochemical characterization of OER and HER were tested on a standard three-electrode cell at the CHI 750 potentiostat. To prepare catalyst ink, 5 mg of catalyst was added in a mixed solution with 500 uL ethanol, 470 uL deionized water and 30 uL Nafion (5 wt%) and then performed ultrasonic for 30 min.

The OER activity of CoFeP-NC in 1M KOH electrolyte solution was determined by typical three-electrode cell of which the catalyst-loaded commercial carbon cloth (1.0 cm × 0.5 cm, loading: 0.8 mg cm^−2^), graphite rod and Ag/AgCl (KCl saturated) was used as working electrode, counter electrode and reference electrode, respectively. The linear sweep voltammetry (LSV) and cyclic voltammetry (CV) were measured under the conditions of a sweep range of 0 to 1.0 V (RHE vs. Ag/AgCl) and a sweep rate of 10 mV s^−1^. The stability of electrocatalyst was tested by cyclic voltammetry of 1000 cycles. The electrochemical impedance spectroscopy (EIS) was determined at a given potential in the frequency range of 1 × 10^−5^ to 1 × 10^5^ Hz.

Unlike OER measurement, the reference electrode was replaced by saturated calomel electrode (SCE) for HER testing. Additionally, conversion of potential into RHE is calculated by formula: E_RHE_ = E_SCE_ + 0.244 + 0.059 × pH. The electrochemical characterization of HER was tested similar to OER. All of the LSV measurements were performed with 90% iR compensation.

## 3. Results and Discussions

### 3.1. Characterization

**Scheme 1:**
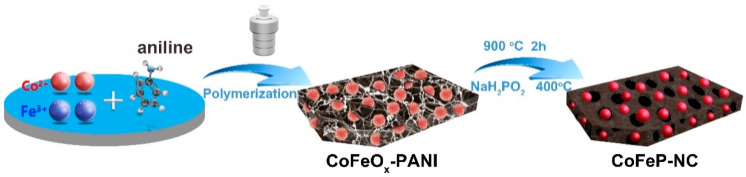
Synthetic schematic diagram of the CoFeP-NC samples.

The synthesis process of CoFeP-NC was illustrated in Scheme 1. The CoFe oxide modified with PANI (CoFeO_x_-PANI) was prepared by one pot hydrothermal method in which Fe^3+^ was acted as oxidant and iron source. After that, the as-prepared CoFeO_x_-PANI was converted to CoFeP-NC through carbonization and phosphorization.

**Figure 1:**
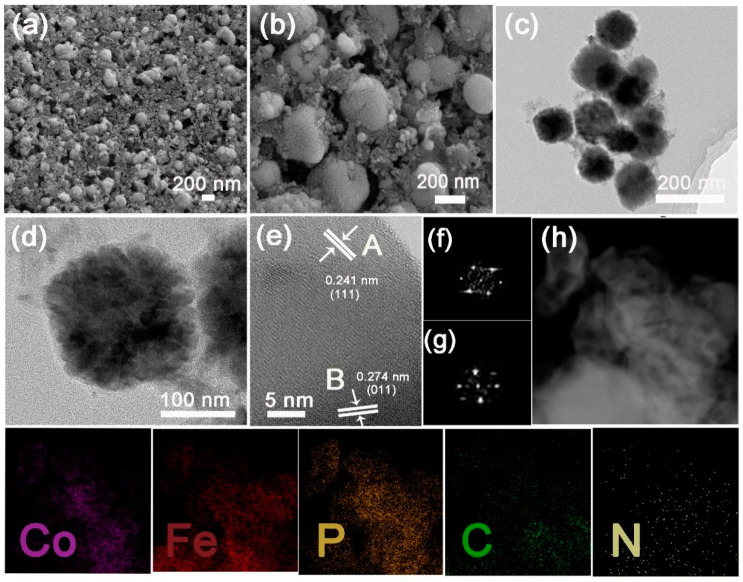
(**a**,**b**) SEM of CoFeP-NC, (**c**,**d**) TEM of CoFeP-NC, (**e**) HRTEM of CoFeP-NC, (**f**,**g**) corresponding fast Fourier-transformed (FFT) patterns for the noted areas of (A, B) in (**e**), respectively. (**h**) TEM image and element distribution of Co, Fe, P, C, and N for CoFeP-NC.

Scanning electron spectroscopy (SEM) and TEM were performed to observe the microstructure of the obtained materials. As shown in the SEM images (Figure 1a,b), the CoFeP is encapsulated by an ultrathin carbon layer and embedded in the carbon network framework. It can be further observed that the uniform size of CoFeP nanospheres is about 150 nm according to Figure 1c,d. HRTEM (Figure 1e) can observe the lattice spacing of 0.274 nm and 0.241 nm, corresponding to the two different crystal planes (011) and (111) of the CoFeP phase, which fully proves the formation of CoFeP. In addition, the corresponding fast Fourier-transformed (FFT) patterns of CoFeP-NC can be revealed in Figure 1f,g. As shown in Figure 1h and Figure S1a, CoFeP-NC was analyzed using energy dispersive X-ray spectroscopy to clarify the elements composition and distribution, and it is proved that the five elements such as Fe and Co are uniformly distributed. The XPS spectra (Figure S1b) further confirmed the electronic state of the composition of CoFeP-NC, indicating the successful preparation of CoFeP-NC.

The morphology of CoFeO_x_-PANI prepared by Fe^3+^-assisted aniline polymerization strategy in different solvents showed different results due to the influence of solution polarity or the dissolution rate of aniline in solution. The SEM images (Figure S2b,e) of the samples synthesized in aqueous solution showed that there was agglomeration but no obvious aniline polymer. In ethanol solution, loose polymerization and slight agglomeration can be observed (Figure S2c,f). It should be noted that when the ratio of ethanol to water is 1:1, CoFeO_x_ nanospheres were surrounded by nanofibers and aniline polymerized tightly (Figure S2a,d). Meanwhile, the corresponding EDX elemental mapping (Figure S2g–k) indicated that elements of Co, Fe, O, C, and N were homogeneously dispersed in CoFeO_x_-PANI. In addition, the result of EDX spectrum (Figure S3a) of CoFeO_x_-PANI was consistent with that of XPS survey spectrum (Figure S3b). In addition, by adjusting the carbonization temperature, CoFe-NC-700, CoFe-NC-800, and CoFe-NC-900 were obtained at 700, 800, and 900 °C, respectively. As shown in Figure S4 CoFe-NC-700 has no obvious change, while the nanofibers on the surface of substrate were partially melt. For CoFe-NC-800, the carbon substrate was obviously cracked, which was benefited for the exposure of active sites. However, when the carbonization temperature was reach 900 ℃, as shown in Figure S4e,f, most of PANI is converted into carbon network framework, while a small part is covered on CoFe. Figure S5 showed the TEM images of CoFeP-NC with different magnification, from which it can be clearly seen that a layer of carbon shell is coated on the surface of the catalyst. The in-situ formed carbon coating on the hybrid nanostructures is conducive to improving its electronic conductivity and accelerating the kinetic process [41] In addition, the diffraction peaks of CoFe crystals remain consistent at different carbonization temperatures (Figure S6), indicating that the crystalline phase did not change during carbonation.

**Figure 2:**
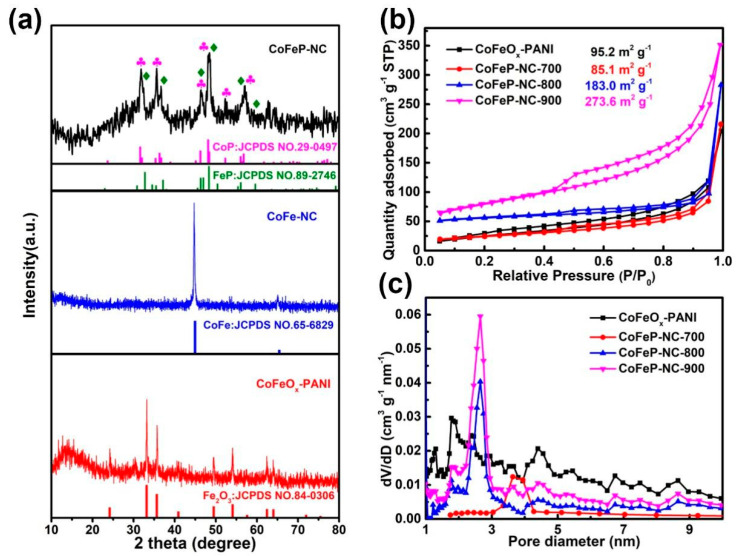
(**a**) XRD patterns of CoFeO_x_-PANI, CoFe-NC, and CoFeP-NC. (**b**) N_2_ adsorption/desorption isotherm and (**c**) pore size distribution of CoFeO_x_-PANI, CoFeP-NC-700, CoFeP-NC-800, and CoFeP-NC-900.

The structures of the materials were further evaluated by XRD. Figure 2a showed the XRD patterns of CoFeO_x_-PANI, CoFe-NC, and CoFeP-NC, respectively. The diffraction peaks of CoFeO_x_-PANI at 24.1°, 33.2°, 35.6°, and 40.9°, which were well corresponded to (012), (104), (110), and (113) planes of Fe_2_O_3_ [42] What is noteworthy is that all diffraction peaks are highly consistent with that of Fe_2_O_3_ (JCPDS NO 84-0306). The absence of other diffraction peaks in XRD pattern implies the formation of amorphous CoO_x_ [43,44]. In addition, the diffraction peaks of CoFe-NC are observed at 44.9° and 65.4°, corresponding to the (110) and (200) phase of CoFe (JCPDS NO 65-6829). It is confirmed that CoFeO_x_ is reduced to CoFe alloy under the effect of PANI. The top of Figure 2a, the existence of diffraction peaks at 32.6°, 37.1°, 46.9°, 48.4°, 56.1°, and 59.6° are attributed to the (011), (111), (202), (211), (212), and (203) reflections, respectively which are consistent with those of FeP (JCPDS No 89-2746) except for the slight shift of diffraction peaks, while the diffraction peaks of CoP at 31.6°, 36.3°, 46.2°, 48.1°, 52.3°, and 56.8°, correspond to the different planes, implying the formation of CoFeP structure (JCPDS NO 29-0497) [45] Compared with CoFe-NC, no diffraction peak of CoFe-NC was observed in CoFeP-NC, demonstrating that CoFe-NC was finally converted to CoFeP-NC after phosphating.

The specific surface area and pore size distributions of CoFeO_x_-PANI, CoFeP-NC-700, CoFeP-NC-800 and CoFeP-NC-900 were evaluated by a Brunauer-Emmett-Teller (BET) measurement. On basis of the N_2_ adsorption/desorption isotherms (Figure 2b), CoFeP-NC-900 showed a specific surface area of 273.6 m^2^ g^−1^, which was higher than that of CoFeO_x_-PANI (95.2 m^2^ g^−1^), CoFeP-NC-700 (85.1 m^2^ g^−1^) and CoFeP-NC-800 (183 m^2^ g^−1^), which means that CoFeP-NC-900 can better expose the active site. And further, as shown in Figure 2c the dominant pore diameter was about 1.6–5.0 nm of CoFeO_x_-PANI, 3.3–4.2 nm of CoFeP-NC-700, 2.8 nm of CoFeP-NC-800 and CoFeP-NC-900. The pore-size distribution indicates the formation of mesopores in the CoFeP-NC samples, which is beneficial to facilitate the access of electrolytes and mass transport during HER and OER process.

**Figure 3:**
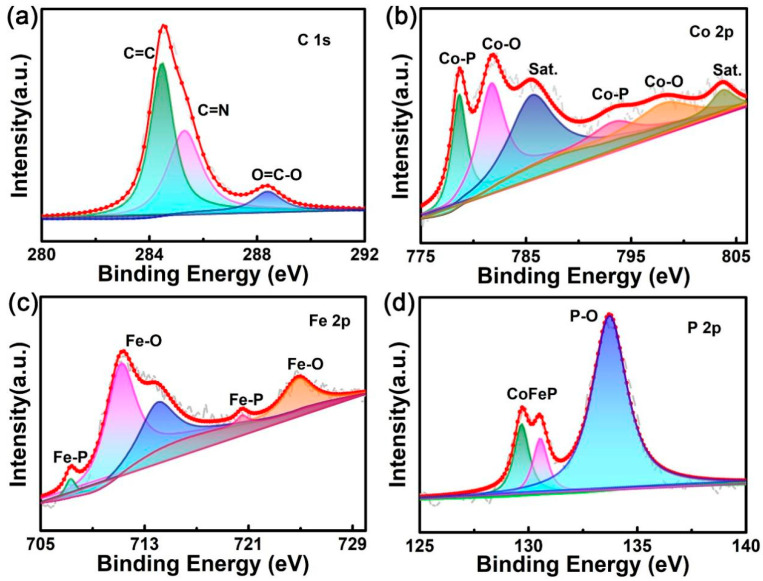
XPS spectrum of (**a**) C 1s, (**b**) Co 2p, (**c**) Fe 2p, and (**d**) P 2p for CoFeP-NC.

In order to understand the molecular structure of the obtained electrocatalyst in detail, the material was further explored by XPS test. Figure 3a showed the high-resolution C 1s spectrum, where the three peaks were observed at 284.5, 285.3 and 288.4 eV, corresponding to C=C, C=N and O=C-O, respectively [46]. In addition, a small amount of N was detected in the CoFeP-NC samples, likely originating from PANI. In the spectrum of N 1s, the corresponding pyrrolic N, graphitic N and pyridinic N can be observed (Figure S7) [47]. For Co 2p (Figure 3b), the existence of two peaks at 778.7 and 793.3 eV can be assigned to Co-P, the peaks at 781.7 and 798.1 eV can be explained as the oxidation form on the catalyst surface and the remaining peaks located at 785.5 and 803.7 eV can be identified as satellite peaks [48] For Fe 2p (Figure 3c), the small peaks located at 707.3 and 720.5 eV can be attributed to the Fe-P. In addition, the oxidized state of Fe species were found at 711.2, 714, and 724.8 eV [49,50]. The spectrum of P 2p was shown in Figure 3d,in which two peaks at 129.7 and 130.5 eV are derived from P 2p_3/2_ and P 2p_1/2_ of FeCoP, while the peak located at 133.7 eV indicated the presence of oxidized P-O species on the surface of FeCoP [51] Herein, through SEM, TEM, XRD, BET, and XPS, a series of characterizations of the composition and microstructure of the material were carried out, and all of the experiments proved the successful preparation of FeCoP-NC.

### 3.2. Electrochemical Characterization

**Figure 4:**
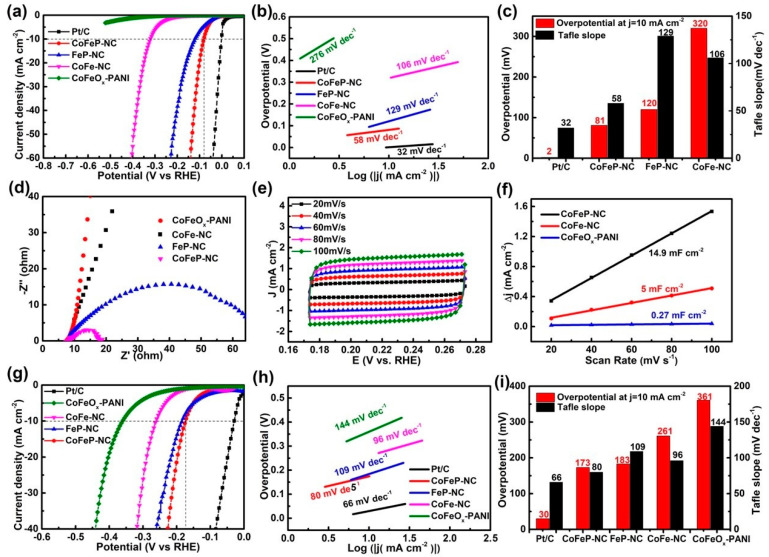
(**a**) Linear sweep voltammetry (LSV) curves, (**b**) Tafel plots, (**c**) overpotentials at η_10_ and Tafel slope value of the as-prepared sample (CoFeP-NC, CoFe-NC, CoFeO_x_-PANI, FeP-NC and RuO_2_) in 0.5 M H_2_SO_4_. (**d**) Nyquist plots of CoFeO_x_-PANI, CoFe-NC, FeP-NC and CoFeP-NC at an applied potential of −0.167 V. (**e**) CVs of CoFeP-NC at different scan rates from 20 to 100 mV s^−1^. (**f**) plots of the capacitive current versus scan rate of CoFeOx-PANI, CoFe-NC and CoFeP-NC. (**g**) LSV curves and (**h**) Tafel plots of CoFeP-NC, CoFe-NC, CoFeO_x_-PANI, FeP-NC, and Pt/C toward HER in 1M KOH. (**g**) LSV curves, (**h**) Tafel plots, (**i**) overpotentials at η_10_ and Tafel slope value of the as-prepared sample (CoFeP-NC, CoFe-NC, CoFeO_x_-PANI, FeP-NC, and RuO_2_) in 1M KOH.

Firstly, HER electrocatalytic activity of CoFeP-NC was examined in 0.5 M H_2_SO_4_ electrolyte at home temperature. Figure 4a–c showed the linear sweep voltammetry (LSV, IR corrected) polarization curves and Tafel slopes of CoFeP-NC, CoFe-NC, CoFeO_x_-PANI, FeP-NC, and 20% Pt/C toward HER. As state-of-art electrocatalyst, the smaller overpotential to drive the η_10_ means the better electrocatalytic property of HER. Compared with FeP-NC, CoFeO_x_-PANI, and CoFe-NC, CoFeP-NC exhibited a lower overpotential, indicating its extraordinary catalytic activity toward HER. Thereinto, the overpotential of CoFeP-NC was 81 mV, which was smaller than the value of FeP-NC (120 mV) and CoFe-NC (320 mV). The Tafel slope is an indispensable parameter for studying the reaction mechanism of the electrocatalysts obtained. Hence, the corresponding LSV curve was transformed into Tafel slope (Figure 4b) by the Tafel equation [20] As shown in Figure 4c, CoFeP-NC showed a small Tafel slope (58 mV dec^−1^), except Pt/C (32 mV dec^−1^), compared with the CoFe-NC (106 mV dec^−1^) and the FeP-NC (129 mV dec^−1^), which demonstrated its favorable kinetics for HER in acid electrolyte. Electrochemical impedance spectroscopy (EIS) was also performed on the as-prepared samples under the same potential conditions to further investigate their reaction kinetics of HER. As shown in Figure 4d, the Nyquist plots of CoFeO_x_-PANI, CoFe-NC, FeP, and CoFeP-NC were obtained by EIS measurements aiming to explore the charge transfer resistance of catalyst–electrolyte interface. Compared with other samples, CoFeP-NC possessed a smaller semicircle, indicating the lower total resistance for HER. The available active sites were further evaluated by exploring the electrochemical active surface area (ECSA) of electrocatalysts. In general, the double-layer capacitance (C_dl_) measured in the non-faradic range is linearly proportional to ECSA. Cyclic voltammetry (CV) Curve of CoFeO_x_-PANI, CoFe-NC, and CoFeP-NC in the voltage range of 0.1–0.3V (vs RHE) with different scan rates (20–120 mV s^−1^) is shown in Figure 4e, Figure S8. The C_dl_ value of CoFeP-NC is 14.9 mF cm^−2^ (Figure 4f), which is larger than that of CoFe-NC (5 mF cm^−2^) and CoFeO_x_ -PANI (0.27 mF cm^−2^), so it has a higher ECSA and more active sites are exposed

For the evaluation of electrocatalyst, durability is an essential parameter in practical application. In order to explore the stability of CoFeP-NC, continuous CV scanning was carried out at the potential range of 0.17–0.27 V (vs RHE). As illustrated in Figure S9a, the two polarization curves of CoFeP-NC showed slightly change after 1000 potential cycles, indicating its stable catalytic capacity. For further verification, the stability of prepared electrocatalyst was measured in 0.5 M H_2_SO_4_. The inset of Figure S9a showed that CoFeP-NC maintained a high stable current density after long-term test in acid electrolyte, further demonstrating the prepared electrocatalyst possessed excellent stable property.

For practical application, the electrocatalytic performance of as-prepared catalysts for HER in 1M KOH were further evaluated. As shown in Figure 4g, CoFeP-NC showed the optimal performance, except commercial Pt/C toward HER in 1 M KOH. In general, Tafel plots was investigated to describe the HER kinetics of catalysts in alkaline medium. As illustrated in Figure 4h,i, the overpotential at current density of 10 mA cm^−2^ for CoFeP-NC was 173 mV and Tafel slope value was 80 mV dec^−1^, which were superior to the other as-prepared catalysts. In addition, CoFeP-NC showed a lower EIS value (Figure S10), indicating the higher charge transfer rate in alkaline electrolyte. After 1000 cycles of continuous CV scanning, the polarization curve of CoFeP-NC exhibited slightly changed (Figure S9b). And the durability of the catalyst was conducted with a constant overpotential. As shown in Figure S9b, the current density maintained well after 10 h of tests, demonstrating CoFeP-NC possessed excellent stable property.

**Figure 5:**
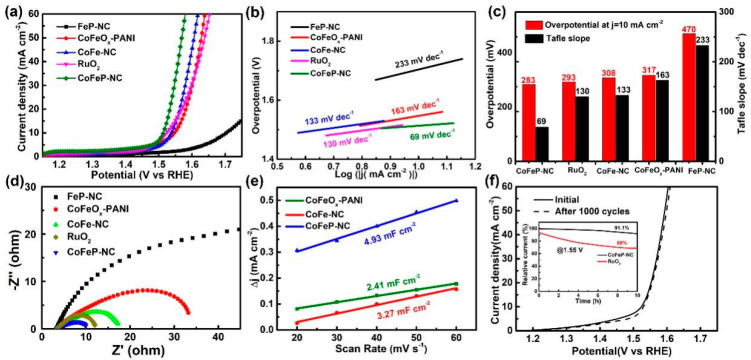
(**a**) LSV curves, (**b**) Tafel plots, (**c**) overpotentials at η_10_ and Tafel slope value of the as-prepared sample (CoFeP-NC, CoFe-NC, CoFeO_x_-PANI, FeP-NC, and RuO_2_). (**d**) Nyquist plots of CoFeO_x_-PANI, CoFe-NC, FeP-NC, RuO_2_, and CoFeP-NC in 1 M KOH at an applied potential of 1.56 V. (**e**) Plots of the capacitive current versus scan rate of CoFeO_x_-PANI, CoFe-NC, and CoFeP-NC. (**f**) The polarization curve for the CoFeP-NC before and after durability test and Chronoamperometric response of CoFeP-NC and RuO_2_.

Besides HER, the electrocatalytic property toward OER was further evaluated, due to the sluggish reaction kinetics. As observed in Figure 5a, CoFeP-NC exhibited the best electrocatalytic activity than the other as-prepared catalysts, even the RuO_2_. A smaller overpotential of CoFeP-NC (283 mV), was needed for CoFe-NC (308 mV), CoFeO_x_-PANI (317 mV), and FeP-NC (470 mV), which was relative to RuO_2_ (293 mV), implying the existence of metallic phase and phosphide played significant role in enhancing OER. Among CoFeP-NC catalysts with different carbonization temperature (Figure S11), CoFeP-NC-900 showed the best OER capability with the lowest overpotential of 283 mV. Notably, the comparison between CoFeP-NC with recently reported materials for OER was listed in Table S2, which was further showed the excellent OER property of CoFeP-NC. As shown in Figure 5b,c, CoFeP-NC presented the smallest Tafel slope of 69 mV dec^−1^, compared with that of RuO_2_ (130 mV dec^−1^), CoFe-NC (133 mV dec^−1^), CoFeO_x_-PANI (163 mV dec^−1^) and FeP-NC (233 mV dec^−1^), indicating CoFeP-NC owned the best reaction kinetics towards OER. EIS was further performed to evaluate ion-transport kinetics of the as-prepared products, and results were shown in Figure 5d. Compared with other samples, CoFeP-NC showed a smaller semicircle, which revealed that the faradaic process of CoFeP-NC hybrid interface was faster. For details, ESCA analysis was exhibited in Figure S12, Figure 5e, and CoFeP-NC (4.93 mF cm^−2^) possessed a larger C_dl_ value than CoFeO_x_-PANI (2.41 mF cm^−2^) and CoFe-NC (3.27 mF cm^−2^) in the non-Faradaic region, demonstrating that CoFeP-NC possessed more active sites. The durability of CoFeP-NC was investigated by continuous CV scanning from 1.2 to 1.7 V in 1 M KOH. After 1000 CV scan cycles, the current density of CoFeP-NC maintained well (Figure 5f). Besides, stability of RuO_2_ and CoFeP-NC were also measured at overpotential of 320 mV, respectively. The current density of CoFeP-NC preserved 91.1% of initial current density after testing 10 h, confirming its outstanding stability for OER. After long-term stability of 10 h, the characteristic peaks of Co-P and Fe-P in high-resolution of P 2p were disappeared (Figure S13a–c), of which the P 2p peaks assigned to FeCoP were disappeared, except phosphate, which was consistent with the change of high-resolution of Co 2p and Fe 2p. Furthermore, XRD patterns of CoFeP-NC showed that there was no obvious characteristic peak after testing (Figure S13d), indicating that the catalyst was transformed into amorphous state due to the in-situ conversion of metal phosphates on the surface to oxides or hydroxides during OER condition [49,52,53].

**Figure 6:**
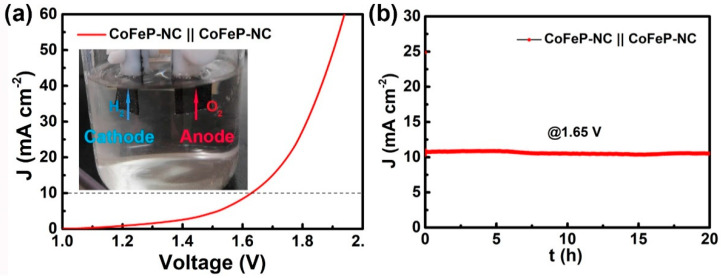
(**a**) LSV curves for CoFeP-NC || CoFeP-NC electrolyzers in alkaline electrolyte (pH = 14) in a two-electrode system. (**b**) Chronoamperometric response of CoFeP-NC || CoFeP-NC.

As mentioned above, the obtained CoFeP-NC showed satisfactory electrocatalytic property. Hence, the sustainable water splitting property of CoFeP-NC was investigated by a two-electrode setup with CoFeP-NC-modified carbon cloth (1.5 mg cm^−2^) as both anode and cathode in alkaline electrolytes. As shown in Figure 6a, CoFeP-NC only needs a low potential of 1.62 V at η_10_. In the process of continuous water electrolysis, electrons are transferred from the anode to the cathode, and these electrons offer electricity to produce O_2_ and H_2_, respectively (inset). The long-term water splitting reaction was carried out under the conditions of 1M KOH and a battery voltage of 1.65V. The current density of water splitting can be remained constant after 20 h (Figure 6b), demonstrating that CoFeP-NC electrocatalyst was promising for practical application.

## 4. Conclusions

In summary, an efficient electrocatalyst CoFeP-NC was developed via carbonation and phosphating treatment for CoFeO_x_-PANI precursor. In this architecture, ultrathin carbon coated CoFeP nanospheres with high activity were uniformly distributed on the porous carbon framework. In alkaline electrolytes, the catalyst exhibits excellent property on both HER and OER, and the overpotential generated when a current density of 10 mA cm^−2^ is achieved in acidic media is 81 mV. The outstanding electrocatalytic property of CoFeP-NC mainly stems from the strong synergistic effect between CoFeP and carbon network framework, which can not only avoid aggregation of CoFeP nanospheres, afford abundant active sites, but also create network framework to offer electronic conductivity and accelerate the access of electrolyte. Our work present a facile and scalable strategy to synthesize efficient and inexpensive electrocatalysts with bifunctional catalytic effects.

## Data Availability

All data reported in this paper is contained within the manuscript.

## Supplementary Materials

The following are available online at https://www.mdpi.com/1996-1944/14/6/1473/s1, Table S1: The comparison of HER catalytic performance between CoFeP-NC and other metal phosphides reported in the literature in 0.5 M H_2_SO_4_, Table S2:The OER catalytic performance of CoFeP-NC in 1 M KOH was compared with that reported recently, Figure S1: (a) EDX spectrum of CoFeP-NC and (b) XPS survey spectrum, Figure S2: SEM images of (a,d) CoFeO_x_-PANI (water / ethanol), (b, e) CoFeO_x_-PANI (water) and (c,f) CoFeO_x_-PANI (ethanol). (g–k) EDX elemental mapping images for CoFeO_x_-PANI, Figure S3: (a) EDX spectrum of CoFeO_x_-PANI and (b) XPS survey spectrum, Figure S4: (a,b) SEM images of CoFe-NC-700 (c,d) SEM images of CoFe-NC-800. (e,f) SEM images of CoFe-NC-900, Figure S5: TEM images of (a–c) CoFe-NC, Figure S6: XRD patterns of CoFe-NC-700, CoFe-NC-800 and CoFe-NC-900, Figure S7: XPS spectrum of N 1 s for CoFeP-NC, Figure S8: CVs of (a) CoFe-NC and (b) CoFeO_x_-PANI at different scan rates from 20 to 100 mV s^−1^ in 0.5 M H2SO4, Figure S9: (a,b) The polarization curve for the CoFeP-NC before and after 1000 cycles in 0.5 M H_2_SO_4_ and in 1 M KOH, respectively. The insets in (a,b) show long term electrolysis curves for CoFeP-NC at overpotentials of 90 and 180 mV, respectively, Figure S10: Nyquist plots of CoFeO_x_-PANI, CoFe-NC, FeP-NC and CoFeP-NC in KOH, Figure S11: LSV curves of CoFe-NC-700, CoFe-NC-800 and CoFe-NC-900, Figure S12: CVs of (a) CoFeP-NC, (b) CoFe-NC and (c) CoFeO_x_-PANI at different scan rates from 20 to 60 mV s^−1^ for OER in 1M KOH, Figure S13: (a) Co 2p, (b) Fe 2p, and (c) P 2p XPS spectra of CoFeP-NC after OER catalysis. (d) XRD images of CoFeP-NC after OER.
