# Supplementary material for: Fe(III) Ions-Assisted Aniline Polymerization Strategy to Nitrogen-Doped Carbon-Supported Bimetallic CoFeP Nanospheres as Efficient Bifunctional Electrocatalysts toward Overall Water Splitting
Source: Materials (Basel). 2021 Mar 17;14(6):1473. doi: 10.3390/ma14061473 (PMC8002635; doi:10.3390/ma14061473)
Supplement: Supplementary file 1 [file materials-14-01473-s001.pdf]

materials

MDPI

Article

Fe(III) Ions-Assisted Aniline Polymerization Strategy to Nitrogen-Doped Carbon-Supported Bimetallic CoFeP Nanospheres as Efficient Bifunctional Electrocatalysts toward Overall Water Splitting

Changhao Zhao [1], Fen Wei [1], Haolin Lv [1], Dengke Zhao [2], Nan Wang [3,*], Ligui Li [2,4,*], Nanwen Li [5] and Xiufang Wang [1,*]

[1] Guangdong Engineering & Technology Research Center of Topic Precise Drug Delivery System, School of Pharmacy, Guangdong Pharmaceutical University, 280 Waihuan Dong Road, University Town, Guangzhou 510006 China; tzzhaochanghao@163.com (C.Z.); wfweifen@163.com (F.W.); lyuhawlin@163.com (H.L.)
[2] Guangzhou Key Laboratory for Surface Chemistry of Energy Materials, New Energy Research Institute, School of Environment and Energy, South China University of Technology, Guangzhou 510006, China; scutezhao@sina.com
[3] Guangdong Provincial Key Laboratory of Optical Fiber Sensing and Communications, Siyuan laboratory, Guangzhou Key Laboratory of Vacuum Coating Technologies and New Energy Materials, Guangdong Provincial Engineering Technology Research Center of Vacuum Coating Technologies and New Energy Materials, Department of Physics, Jinan University, Guangzhou, Guangdong 510632, China.
[4] Guangdong Provincial Key Laboratory of Advance Energy Storage Materials, South China University of Technology, Guangzhou, 510640, China
[5] Key Laboratory of Coal Conversion, Institute of Coal Chemistry, Chinese Academy of Sciences, Taiyuan 030001, China; linanwen@sxicc.ac.cn
* Correspondence: esguili@scut.edu.cn (L.L.); nanwang@jnu.edu.cn (N.W.); x_f_wang@163.com (X.W.)

Table S1. The comparison of HER catalytic performance between CoFeP-NC and other metal phosphides reported in the literature in 0.5 M H_2SO_4.

Catalyst	Current density (mA cm^{-2})	η_{10} (mV)	Tafel slope (mV dec^{-1})	Reference
CoP/CSs-0.05	10	94	60	1
CoP/CN@MoS_2	10	144	69	2
Co-FeP/Ti	10	126	64	3
FeP/CP	10	140	62	4
FeP- Ⅰ NS	10	95	41	5
MoP/N, P-rGO	10	115	54	6
S-MoP NPL	10	86	34	7
$Fe_{0.074}$NiP/NWM	10	108	52	8
NiCoP/CNTs	10	267	88	9
FeCoP-NC	10	81	58	This work

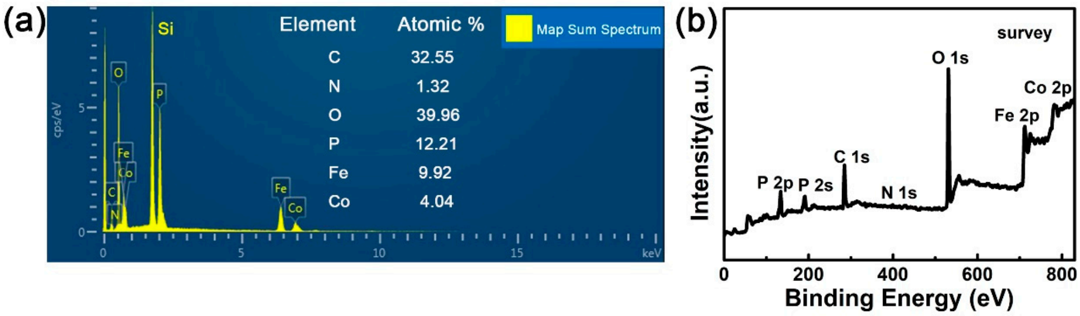

Figure S1. (**a**) EDX spectrum of CoFeP-NC and (**b**) XPS survey spectrum.

Table S2. The OER catalytic performance of CoFeP-NC in 1 M KOH was compared with that reported recently.

Catalyst	Current density (mA cm^{-2})	η_{10} (mV)	Tafel slope (mV dec^{-1})	Reference
CoP-TiO_x	10	337	72	10
CoPS@C	10	313	90	11
CoP/NC-400	10	320	89	12
porous Ni_2P	10	320	105	13
NiCoP/C@FeOOH	10	271	69	14
Fe-NiCoP/PBA HNCs	10	290	70	15
V-CoP	10	340	96	16
CoNiP/NC700	10	300	66	17
CoNiP NWs	10	301	54	18
CoFeP-NC	10	283	64	This work

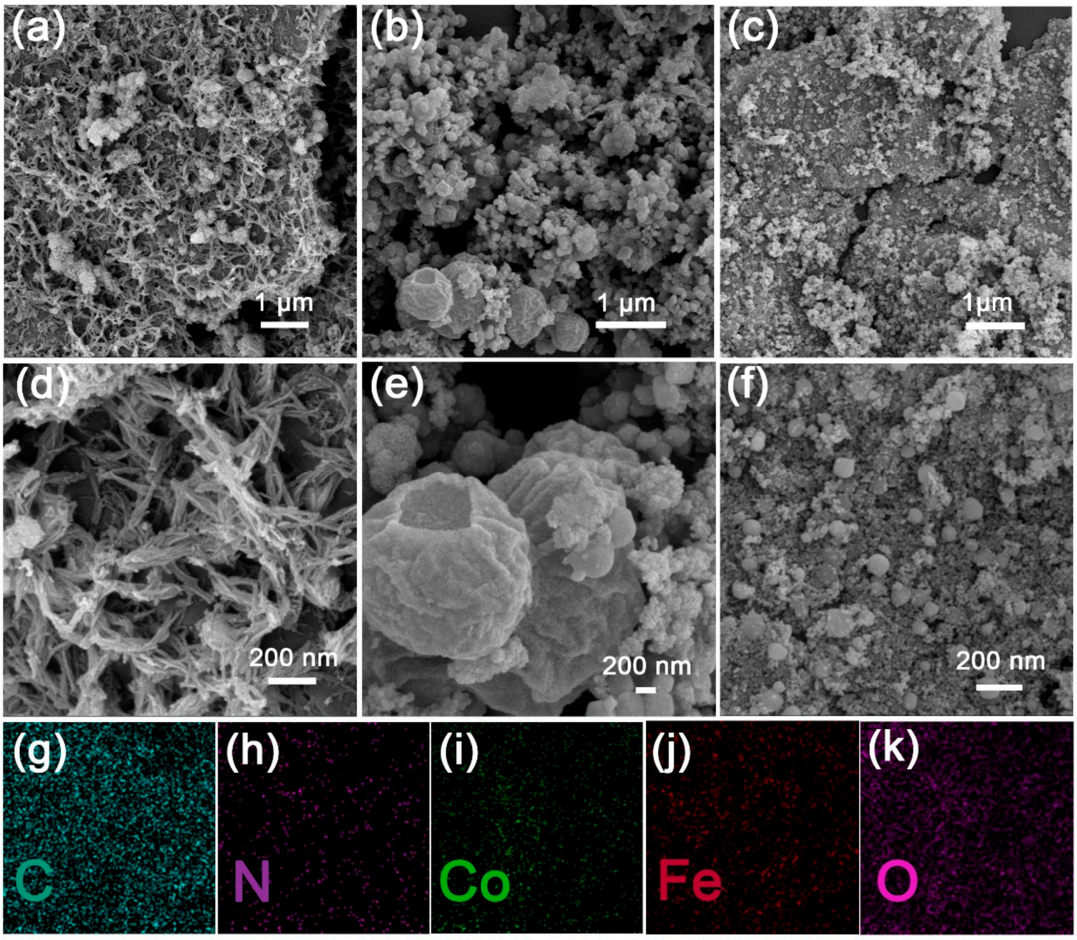

Figure S2. SEM images of (**a, d**) $CoFeO_x$-PANI (water / ethanol), (**b, e**) $CoFeO_x$-PANI (water) and (**c, f**) $CoFeO_x$-PANI (ethanol). (**g-k**) EDX elemental mapping images for $CoFeO_x$-PANI.

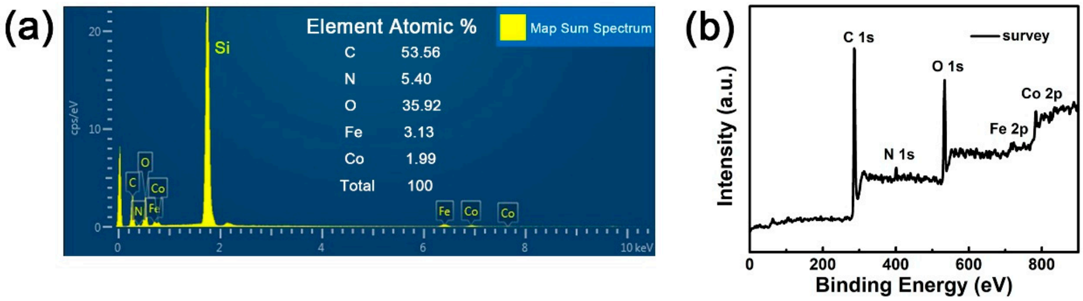

Figure S3. (**a**) EDX spectrum of $CoFeO_x$-PANI and (**b**) XPS survey spectrum.

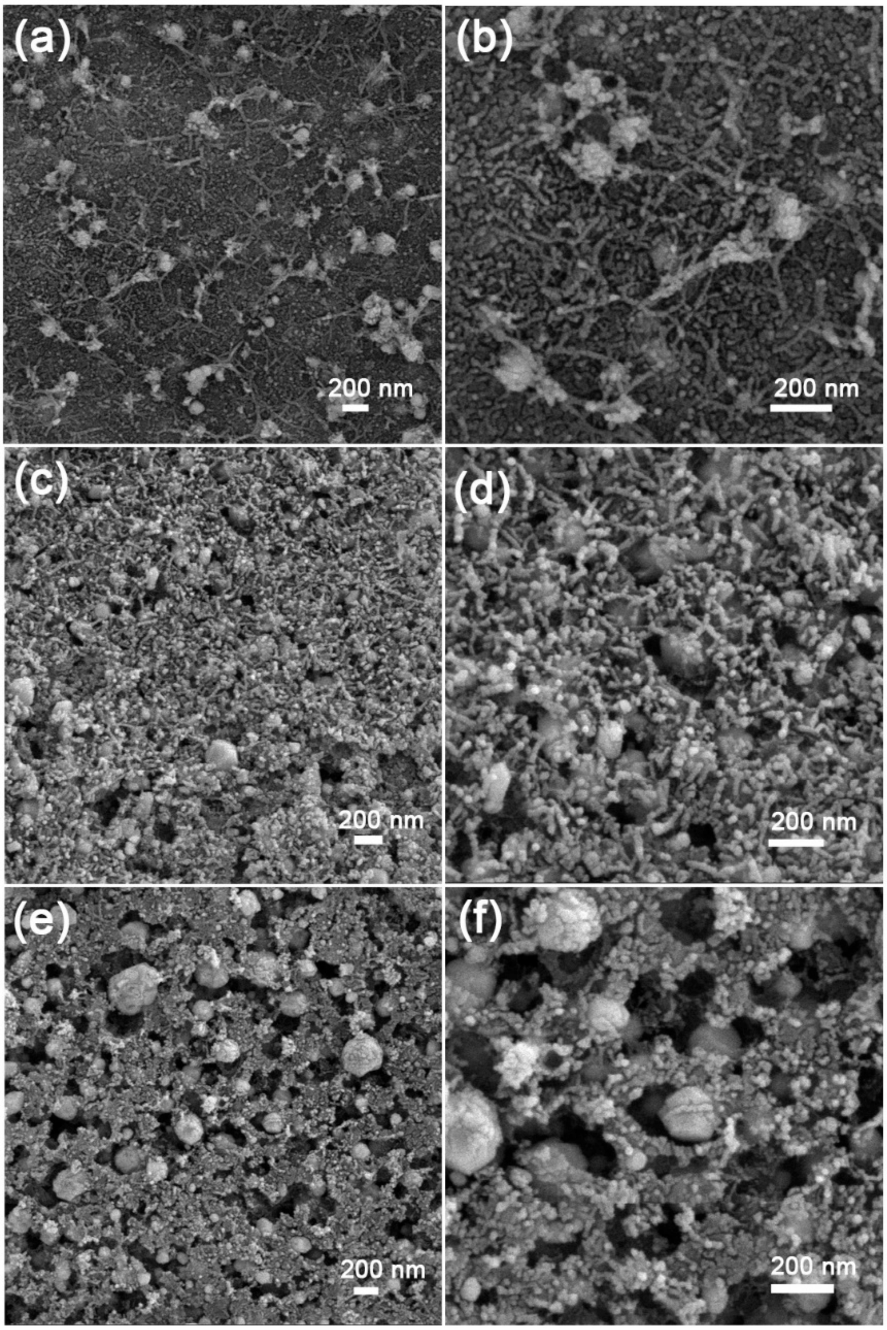

Figure S4. (**a, b**) SEM images of CoFe-NC-700.(**c, d**) SEM images of CoFe-NC-800. (e, f) SEM images of CoFe-NC-900.

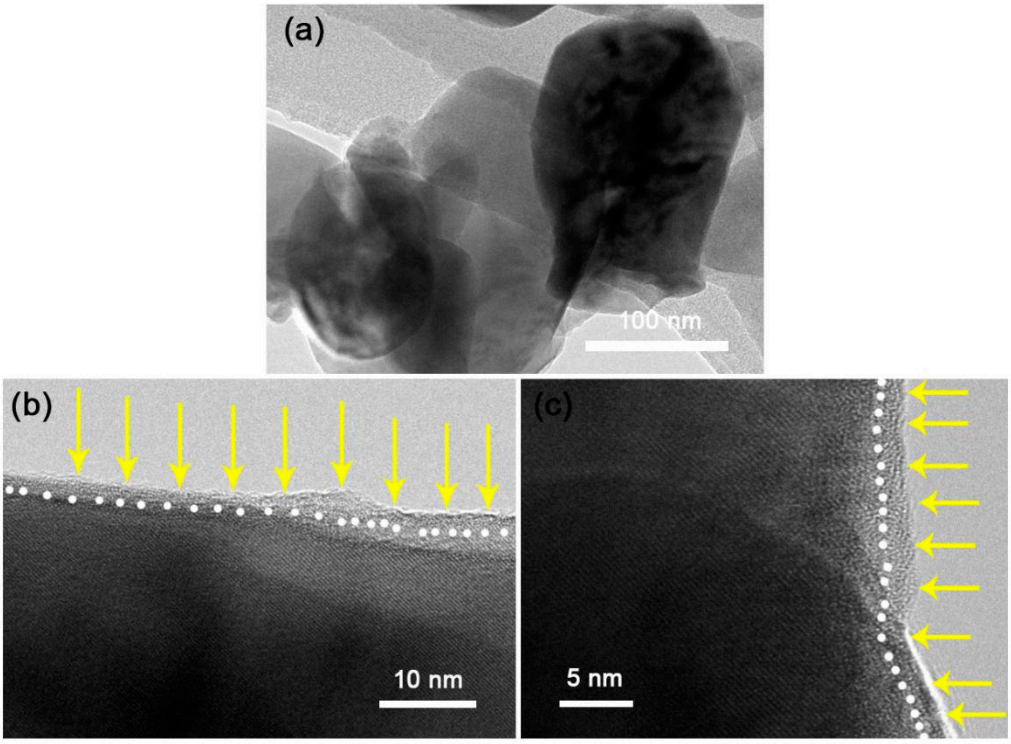

Figure S5. TEM images of (**a, b, c**) CoFe-NC.

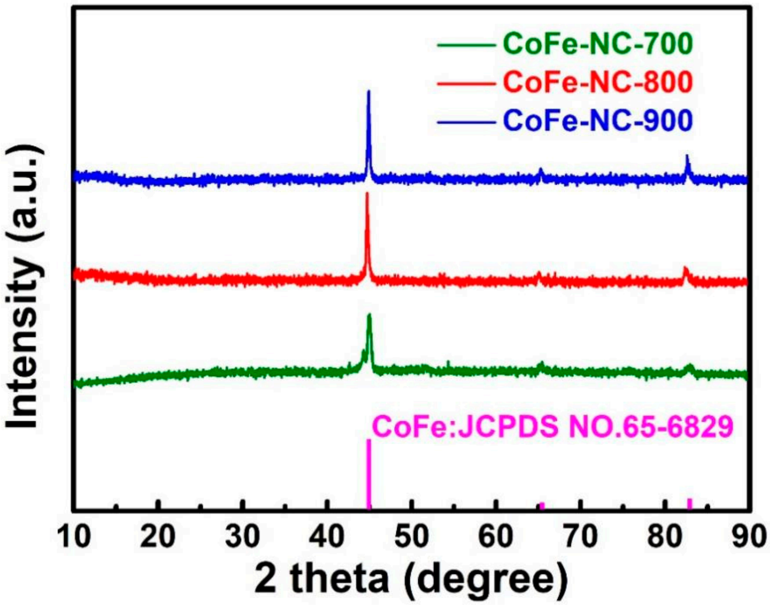

Figure S6. XRD patterns of CoFe-NC-700, CoFe-NC-800 and CoFe-NC-900.

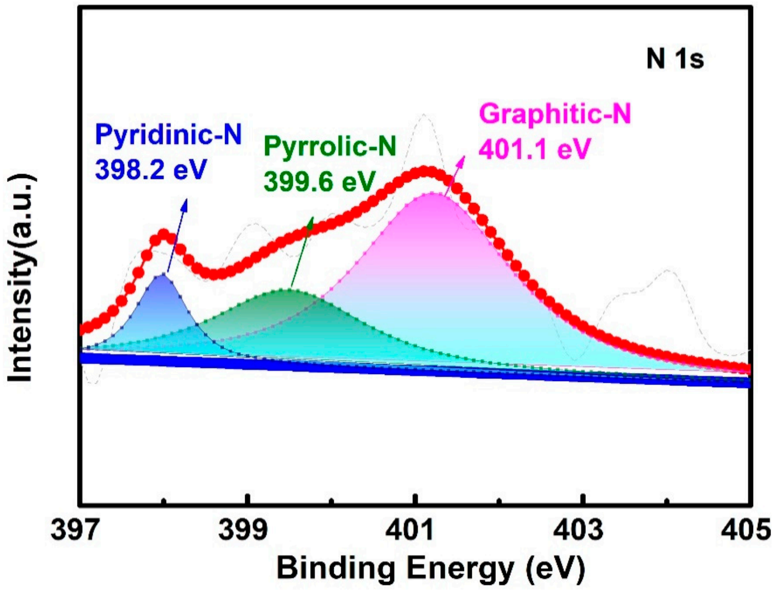

Figure S7. XPS spectrum of N 1s for CoFeP-NC.

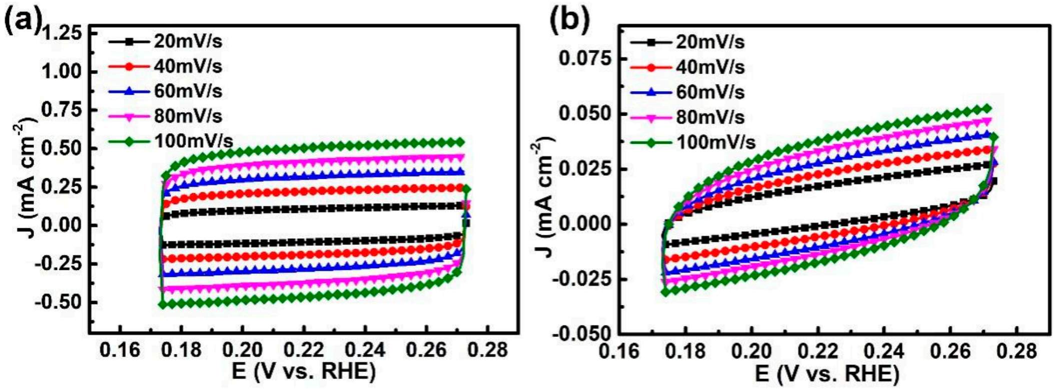

Figure S8. CVs of (**a**) CoFe-NC and (**b**) $CoFeO_x$-PANI at different scan rates from 20 to 100 mV s^{-1} in 0.5 M H_2SO_4.

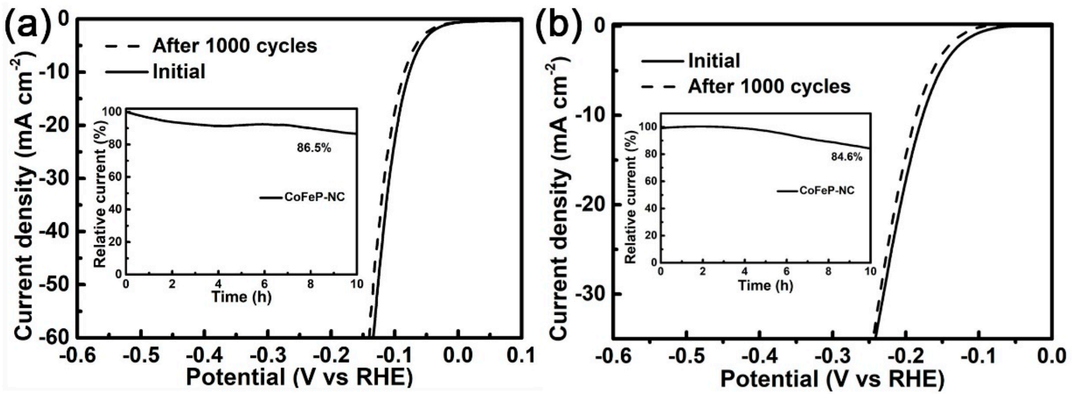

Figure S9. (**a, b**) The polarization curve for the CoFeP-NC before and after 1000 cycles in 0.5 M H_2SO_4 and in 1 M KOH, respectively. The insets in (**a, b**) show long term electrolysis curves for CoFeP-NC at overpotentials of 90 and 180 mV, respectively.

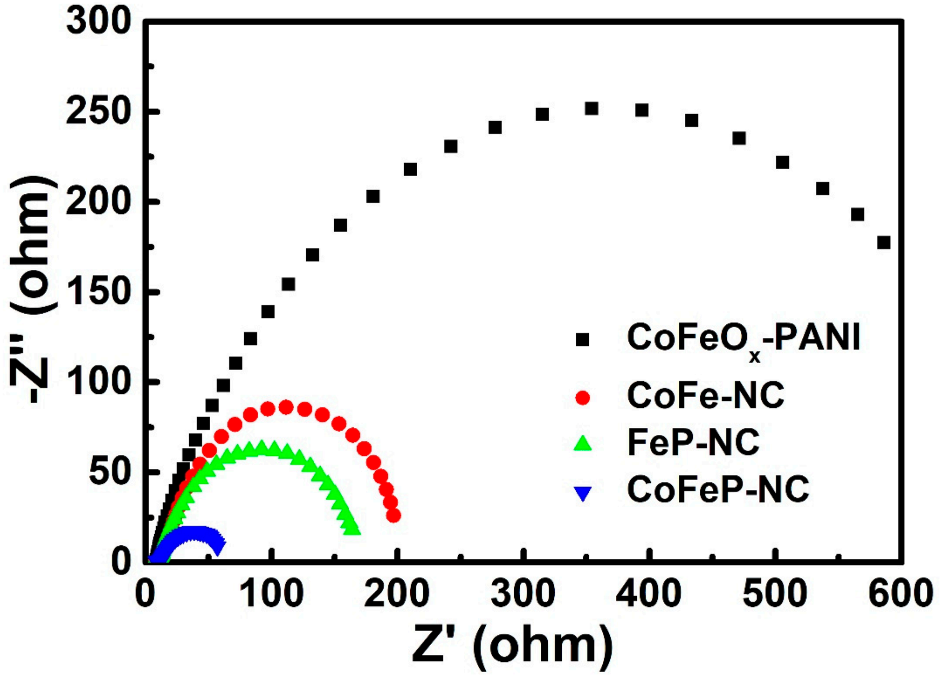

Figure S10. Nyquist plots of $CoFeO_x$-PANI, CoFe-NC, FeP-NC and CoFeP-NC in KOH.

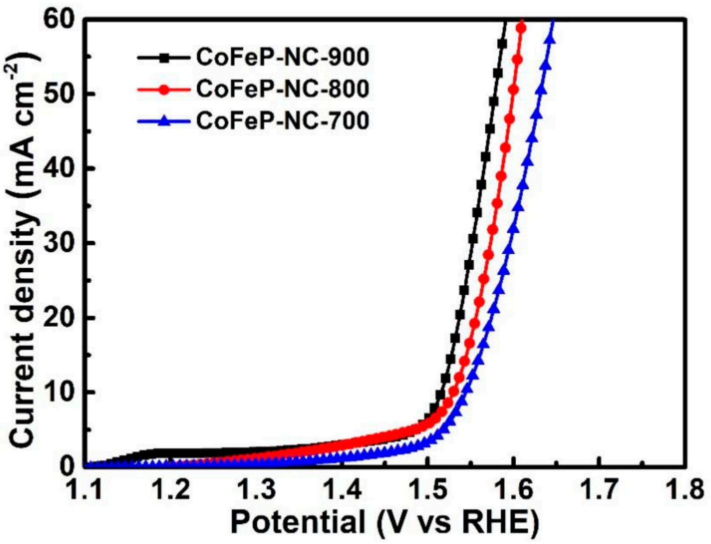

Figure S11. LSV curves of CoFe-NC-700, CoFe-NC-800 and CoFe-NC-900.

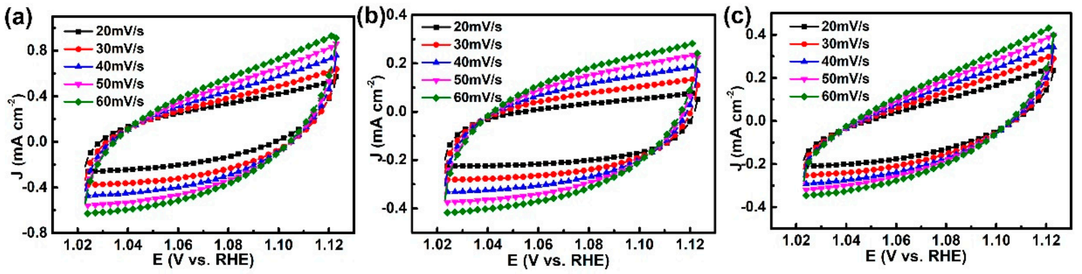

Figure S12. CVs of (**a**) CoFeP-NC, (**b**) CoFe-NC and (**c**) $CoFeO_x$-PANI at different scan rates from 20 to 60 mV s^{-1} for OER in 1M KOH.

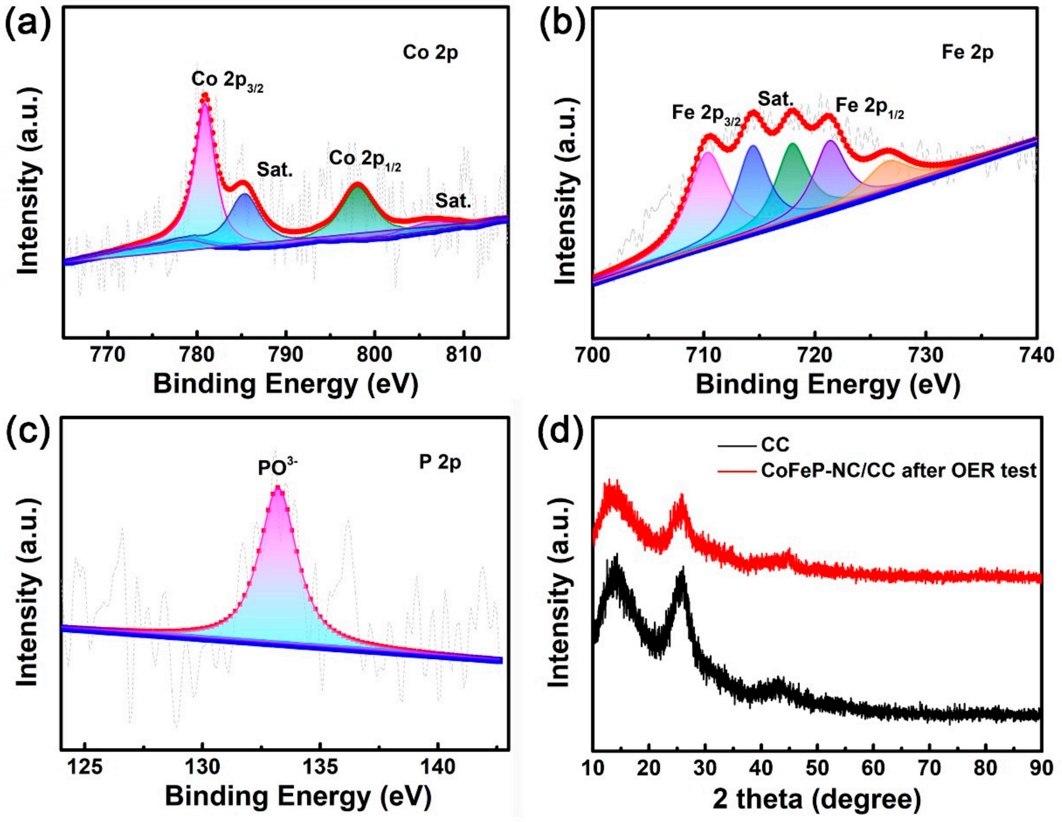

Figure S13. (**a**) Co 2p, (**b**) Fe 2p, and (**c**) P 2p XPS spectra of CoFeP-NC after OER catalysis. (**d**) XRD images of CoFeP-NC after OER.

References

1. Gao, S.; Yang, M.; Li, S.; Xia, J.; Mei, J.; Xie, S.; Liu, H. A phosphorus-doped carbon sphere supported CoP nanocatalyst for electrochemical hydrogen evolution. *Sustainable Energy & Fuels* **2019**, *3*, 823–830.
2. Li, J.G.; Xie, K.; Sun, H.; Li, Z.; Ao, X.; Chen, Z.; Ostrikov, K.K.; Wang, C.; Zhang, W. Template-Directed Bifunctional Dodecahedral CoP/CN@MoS_2 Electrocatalyst for High Efficient Water Splitting. *ACS Appl Mater Interfaces* **2019**, *11*, 36649–36657.
3. Cho, G.; Park, Y.; Kang, H.; Hong, Y.-k. Lee, T.; Ha, D.-H. Transition metal-doped FeP nanoparticles for hydrogen evolution reaction catalysis. *Applied Surface Science* **2020**, *510*, 145427.
4. Shi, J.; Qiu, F.; Yuan, W.; Guo, M.; Yuan, C.; Lu, Z-H., Novel electrocatalyst of nanoporous FeP cubes prepared by fast electrodeposition coupling with acid-etching for efficient hydrogen evolution.. *Electrochimica Acta* **2020**, *329*, 135185.
5. Zhang, X.; Ji, J.; Yang, Q.; Zhao, L.; Yuan, Q.; Hao, Y.; Jin, P.; Feng, L. Phosphate Doped Ultrathin FeP Nanosheets as Efficient Electrocatalysts for the Hydrogen Evolution Reaction in Acid Media. *ChemCatChem* **2019**, *11*, 2484–2489.
6. Zhang, J.; Wang, X.; Xue, Y.; Xu, Z.; Pei, J.; Zhuang, Z. Self-assembly precursor-derived MoP supported on N, P-Codoped reduced graphene oxides as efficient catalysts for hydrogen evolution reaction. Inorg. Chem. **2018**, *57*, 13859–13865.

7. Liang, K.; Pakhira, S.; Yang, Z.; Nijamudheen, A.; Ju, L.; Wang, M. Aguirre-Velez, C.I.; Sterbinsky, G.E.; Du, Y.; Feng, Z. Mendoza-Cortes, J.L.; Yang, Y. S-Doped MoP Nanoporous Layer Toward High-Efficiency Hydrogen Evolution in pH-Universal Electrolyte. *ACS Catalysis* **2018**, *9*, 651–659.
8. Hui, B.; Li, J.; Lu, Y.; Zhang, K.; Chen, H.; Yang, D.; Cai, L.; Huang, Z. Boosting electrocatalytic hydrogen generation by a renewable porous wood membrane decorated with Fe-doped NiP alloys. *Journal of Energy Chemistry* **2021**, *56*, 23–33.
9. Wang, Q.; Hou, M.; Huang, Y.; Li, J.; Zhou, X.; Ma, G.; Ren, S. One-pot synthesis of NiCoP/CNTs composites for lithium ion batteries and hydrogen evolution reaction. *Ionics* **2019**, *26*, 1771–1778.
10. Liang, Z.; Zhou, W.; Gao, S.; Zhao, R.; Zhang, H.; Tang, Y.; Cheng, J.; Qiu, T.; Zhu, B.; Qu, C.; Guo, W.; Wang, Q.; Zou, R. Fabrication of Hollow CoP/TiO_x Heterostructures for Enhanced Oxygen Evolution Reaction. *Small* **2020**, *16*, 1905075.
11. Wang, M.; Tang, K. Enhanced electrochemical properties of cellular CoPS@C nanocomposites for HER, OER and Li-ion batteries. *RSC Advances* **2019**, *9*, 14859–14867.
12. Chen, L.; Xu, G.C.; Xu, G.; Zhang, L. CoP/N-Doped Carbon Nanowire Derived from Co-Based Coordination Polymer as Efficient Electrocatalyst toward Oxygen Evolution Reaction. *Energy Technology* **2020**, *8*, 1901419.
13. Wang, Q.; Liu, Z.; Zhao, H.; Huang, H.; Jiao, H.; Du, Y.MOF-derived porous Ni_2P nanosheets as novel bifunctional electrocatalysts for the hydrogen and oxygen evolution reactions. *Journal of Materials Chemistry A* **2018**, *6*, 18720–18727.
14. Li, J.G.; Gu, Y.; Sun, H.; Lv, L.; Li, Z.; Ao, X.; Xue, X.; Hong, G.; Wang, C. Engineering the coupling interface of rhombic dodecahedral NiCoP/C@FeOOH nanocages toward enhanced water oxidation. *Nanoscale* **2019**, *11*, 19959–19968.
15. Li, D.; Liu, C.; Ma, W.; Xu, S.; Lu, Y.; Wei, W.; Zhu, J.; Jiang, D. Fe-doped NiCoP/Prussian blue analog hollow nanocubes as an efficient electrocatalyst for oxygen evolution reaction. *Electrochimica Acta* **2020,** 137492.
16. Qin, J.; Lin, J.; Chen, T.; Liu, Da. Xie, J.; Guo, B.; Wang, L.; Chai, Y.; Dong, B. Facile synthesis of V-doped CoP nanoparticles as bifunctional electrocatalyst for efficient water splitting. *Journal of Energy Chemistry* **2019**, *39*, 182–187.
17. Li, J.; Du, G.; Cheng, X.; Feng, P.; Luo, X. CoNiP/NC polyhedrons derived from cobalt-based zeolitic imidazolate frameworks as an active electrocatalyst for oxygen evolution. *Chinese Journal of Catalysis* **2018**, *39*, 982–987.
18. Amorim, I.; Xu, J.; Zhang, N.; Xiong, D.; Thalluri, S.M.; Thomas, R.; Sousa, J.P.S.; Araújo, A.; Li, H.; Liu, L. Bi-metallic cobalt-nickel phosphide nanowires for electrocatalysis of the oxygen and hydrogen evolution reactions. *Catalysis Today* **2020**, *358*, 196–202.
